# Neoechinulin A as a Promising SARS-CoV-2 M^pro^ Inhibitor: In Vitro and In Silico Study Showing the Ability of Simulations in Discerning Active from Inactive Enzyme Inhibitors

**DOI:** 10.3390/md20030163

**Published:** 2022-02-24

**Authors:** Hani A. Alhadrami, Gaia Burgio, Bathini Thissera, Raha Orfali, Suzan E. Jiffri, Mohammed Yaseen, Ahmed M. Sayed, Mostafa E. Rateb

**Affiliations:** 1 Department of Medical Laboratory Technology, Faculty of Applied Medical Sciences, King Abdulaziz University, P.O. Box 80402, Jeddah 21589, Saudi Arabia; hanialhadrami@kau.edu.sa; 2 Molecular Diagnostic Laboratory, King Abdulaziz University Hospital, King Abdulaziz University, P.O. Box 80402, Jeddah 21589, Saudi Arabia; 3 Special Infectious Agent Unit, King Fahd Medical Research Center, King Abdulaziz University, P.O. Box 80402, Jeddah 21589, Saudi Arabia; 4 School of Computing, Engineering and Physical Sciences, University of the West of Scotland, Paisley PA1 2BE, UK; gaia.burgio@uws.ac.uk (G.B.); bathini.thissera@uws.ac.uk (B.T.); mohammed.yaseen@uws.ac.uk (M.Y.); 5 Department of Pharmacognosy, College of Pharmacy, King Saud University, P.O. Box 22452, Riyadh 11495, Saudi Arabia; rorfali@ksu.edu.sa; 6 King Fahd Medical Research Center, King Abdulaziz University, P.O. Box 80402, Jeddah 21589, Saudi Arabia; suzanaljiffri@gmail.com; 7 Department of Pharmacognosy, Faculty of Pharmacy, Nahda University, Beni-Suef 62513, Egypt

**Keywords:** SARS-CoV-2 M^pro^, neoechinulin A, *Aspergillus fumigatus*, molecular docking, steered molecular dynamics simulation

## Abstract

The COVID-19 pandemic and its continuing emerging variants emphasize the need to discover appropriate treatment, where vaccines alone have failed to show complete protection against the new variants of the virus. Therefore, treatment of the infected cases is critical. This paper discusses the bio-guided isolation of three indole diketopiperazine alkaloids, neoechinulin A (**1**), echinulin (**2**), and eurocristatine (**3**), from the Red Sea-derived *Aspergillus fumigatus* MR2012. Neoechinulin A (**1**) exhibited a potent inhibitory effect against SARS-CoV-2 M^pro^ with IC_50_ value of 0.47 μM, which is comparable to the reference standard GC376. Despite the structural similarity between the three compounds, only **1** showed a promising effect. The mechanism of inhibition is discussed in light of a series of extensive molecular docking, classical and steered molecular dynamics simulation experiments. This paper sheds light on indole diketopiperazine alkaloids as a potential structural motif against SARS-CoV-2 M^pro^. Additionally, it highlights the potential of different molecular docking and molecular dynamics simulation approaches in the discrimination between active and inactive structurally related M^pro^ inhibitors.

## 1. Introduction

For approximately two years, the global pandemic caused by the severe acute respiratory syndrome coronavirus 2 (SARS-CoV-2) has cost many lives and put the world economy on hold in 2020, with extreme consequences. In December 2021, the number of confirmed SARS-CoV-2 cases worldwide reached around 350 million, with more than 5.5 million reported deaths [1]. The world’s focus suddenly shifted to the scientific community, which has diligently delivered several vaccines that positively impacted the new cases and hospitalization. However, apparent imbalances in the world economy are causing a delay in the rolling out of the vaccines, especially in low-income countries, delaying the end of the epidemic and costing thousands of lives each day [2]. Moreover, infections have occurred in some cases despite vaccination, and the continuous announcement of new viral variants that could be resistant to the current vaccines represents an obvious challenge. Hence, finding an effective treatment as a weapon besides the vaccination campaign is still relevant and crucial in tackling the virus.

**Figure 1:**
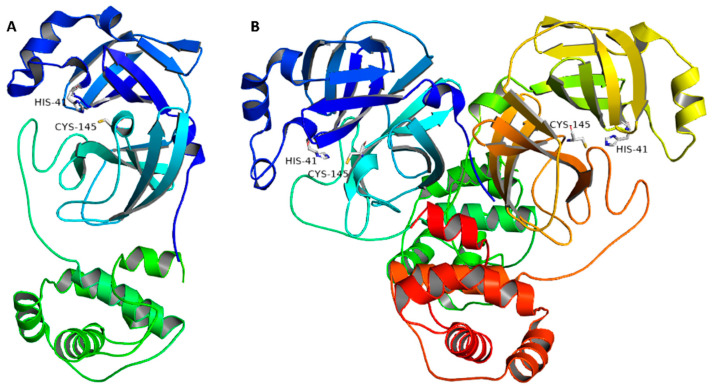
(**A**) Monomeric structure of SARS CoV-2 M^pro^ (PDB code: 7LTJ) showing its three main domains (I, II, and III; blue, cyan, and green, respectively) along with the catalytic dyad (HIS-41:CYS-145). (**B**) The dimeric active form of SARS CoV-2 M^pro^.

Generally, corona viruses’ main proteases occur in dimeric forms, and their activity decreases profoundly if this conserved dimerization is inhibited by mutations. Each monomer consists of three domains (domains I, II, and III), where the catalytic binding site occurs between domains I and II, while domain III is responsible for the enzyme dimerization (Figure 1). The enzyme active site has a conserved HIS-41-CYS-145 catalytic dyad that is essential for peptide cleavage. Hence, any alteration in these catalytic key residues will lead to a complete loss of enzyme activity. A number of covalent inhibitors (i.e., covalent bonding to CYS-145) have been previously reported, while non-covalent competitive inhibitors are much less investigated [3].

Virtual screenings by means of advanced computer-aided programs that perform molecular docking have enabled the screening of thousands of compounds in a short time span, accelerating the discovery of new drug leads [4]. The identification of new drug targets supersedes the development and licensing of new medicines. For example, from 2012 to 2017, only 12 new antivirals were approved by the FDA, of which eight are prescribed for the treatment of hepatitis C virus (HCV), and two are used in combinations with other drugs against the human immunodeficiency virus (HIV). However, the WHO and governments are recurrently challenged by (re)emerging viruses, which cause pandemics with no specific treatment, such as Zika virus (ZIKV), Ebola virus (EBOV), Middle East respiratory syndrome coronavirus (MERS-CoV), and SARS-CoV-2 [5]. Although drug repurposing is one of the main strategies for quick discovery of such treatments, remdesivir was the only antiviral drug approved by the FDA to treat SARS-CoV-2 patients who need hospitalization [6]. Additionally, the European Medicine Agency (EMA) has also approved remdesivir and three other medications [7]. Recently, molnupiravir (Merck Lagevrio^®^) was FDA-approved for oral treatment of COVID-19 non-hospitalized patients with mild-to-moderate symptoms [8] with a note from the European Medicine Agency (EMA) that, although not authorized in the EU, it could be prescribed for adults with COVID-19 who do not need supplemental oxygen or at increased risk of developing severe COVID-19.

Given the crucial role of SARS-CoV-2 M^pro^ in viral replication in addition to the absence of spike protein variant resistance challenges, SARS-CoV-2 M^pro^ inhibition is considered an attractive target for approach for small-molecule oral antiviral therapeutics to treat COVID-19 [9]. Using this target for drug discovery, PF-07321332, an orally bioavailable SARS-CoV-2 M^pro^ inhibitor, showed promising anti-SARS-CoV-2 oral activity in vivo in mouse model as well as excellent oral plasma concentrations in a phase 1 human clinical trial [9]. Moreover, Pfizer’s oral anti-SARS-CoV-2 M^pro^ drug paxlovid showed significant reduction in hospital admissions and deaths among people with COVID-19, when compared with placebo [10], hence it is approved by the FDA and recommended with conditional marketing by the EMA.

Due to their eminent chemical and biological diversity, natural products are considered a promising source for different structural motifs and drug leads. Out of the 1881 new chemical entities (NCEs) approved by the FDA between 1980 and 2019 covering all sources and diseases, approximately 55% are natural products, analogs, or natural product mimics. In the anti-infective areas (including antibacterial, antifungals, and antivirals), 60% of the 415 agents currently available on the market are naturally derived or originated from natural products [11]. Recently, the microbial-derived FDA-approved anti-parasitic drug ivermectin, pentacyclic lactone derived from the soil bacterium *Streptomyces avermitilis*, proved to be effective as an in vitro inhibitor of SARS-CoV-2 replication [12]. Clinical trials assessing ivermectin for the treatment of COVID-19 patients are ongoing.

Our group has reacted immediately with the pandemic, putting every effort to screen our available extracts, fractions, and pure compounds obtained from plants, endophytes, and microorganisms of marine and terrestrial origin for the discovery of antiviral hits against SARS-CoV-2 or its main protease (M^pro^) as a potential target [13,14,15,16,17,18]. In the current study, we report the discovery of a previously reported indole diketopiperazine alkaloid neoechinulin A (**1**), as a promising SARS-CoV-2 M^pro^ inhibitor through bioguided screening. Despite the structural similarity between the isolated compounds, only **1** showed a promising effect. The mechanism of inhibition and the significant difference in the activity are discussed using classical and steered molecular dynamics simulation experiments.

## 2. Results

### 2.1. Bioguided Isolation and Structural Identification

**Figure 2:**
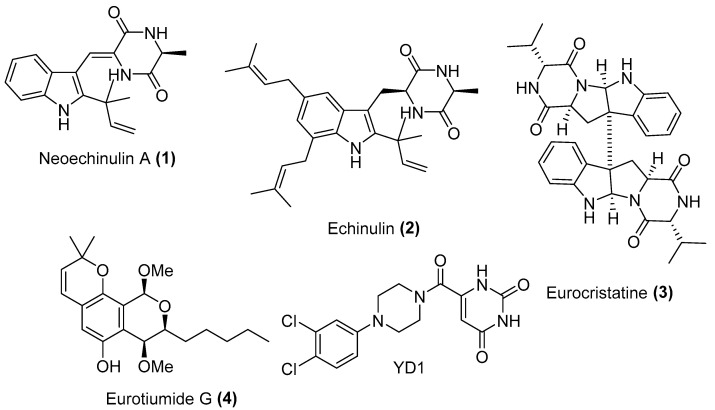
Compounds isolated from *Aspergillus fumigatus* MR2012 and the M^pro^ co-crystalized inhibitor.

Screening of our in-house total of 100 extracts comprising 20 marine and 15 terrestrial fungal extracts cultured on different culture media against SARS-CoV-2 M^pro^ pinpointed the extract of the Red Sea-derived *Aspergillus fumigatus* MR2012 as a potential to follow. Large scale fermentation, extraction, and fractionation with organic solvents with different polarities followed by SARS-CoV-2 M^pro^ screening, indicated the dichloromethane (DCM) as an active fraction. Purification of the HPLC fraction on semi-preparative RP-HPLC led to the isolation of four major compounds (Figure 2). Structure elucidation of the isolated molecules was based on HRESIMS analysis together with ^1^H, ^13^C, and multiplicity-edited HSQC spectra using SMART 2.1 database [19] and The Natural Products Atlas 2.0 [20], in addition to comparison with the literature data. This analysis led to the identification of the isolated metabolites as two prenylated indole diketopiperazines (DKP) neoechinulin A (**1**) [21] and echinulin (**2**) [22], the indole DKP dimer eurocristatine (**3**) [23], and the isocoumarin derivative eurotiumide G (**4**) [24]. The structures of the isolated compounds were further confirmed by COSY and HMBC correlations.

### 2.2. In Vitro Screening against SARS-CoV-2 M^pro^

**Figure 3:**
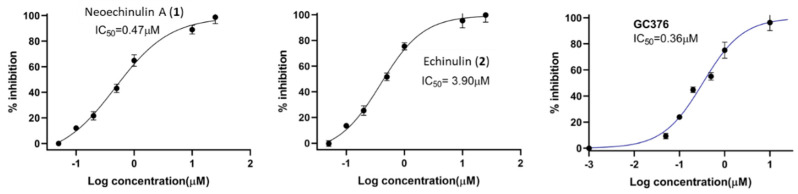
Dose-response curves of compounds **1** and **2**, and the positive control GC376 against SARS-CoV-2 M^pro^.

Based on our initial SARS-CoV-2 M^Pro^ screening, the total methanolic extract and the DCM fraction showed good inhibition when subjected to an in vitro evaluation on the SARS-CoV-2 M^Pro^ using FRET assay with the known inhibitor GC376 as a positive control (data not shown). Evaluation of the four isolated metabolites indicated that only neoechinulin A (**1**) had significant SARS-CoV-2 M^pro^ inhibitory effects with IC_50_ value 0.47 μM compared to GC376 as a positive control with IC_50_ value of 0.36 µM (Figure 3). Echinulin (**2**) showed weak inhibition with an IC_50_ at 3.90 µM, while eurocristatine (**3**) showed weak enzymatic inhibition of less than 50% inhibition at 5 µM concentration, hence its IC_50_ was not calculated. The isocoumarin derivative eurotiumide G (**4**) showed no activity.

### 2.3. Docking and Molecular Dynamics Simulations

To find out how these indole-based natural products can interact and inhibit M^pro^, they were subjected to a series of docking and molecular dynamics simulation-based experiments. Looking into previously reported non-covalent inhibitors, YD1 (i.e., the co-crystalized inhibitor of M^pro^ with PDB code: 7LTJ) was observed to be the inhibitor with the most structural similarities to compounds **1** and **2**. Accordingly, this structure was selected for all of the subsequent in silico-based analyses. Despite using it as a reference inhibitor in the in vitro assays, GC376-contatining M^pro^ model was not selected for the in silico analysis due to its covalent interaction with CYS-145 inside the enzyme active site.

First (docking step), the three compounds were docked into the M^pro^ active site to estimate their fitting score inside this binding pocket. Before initiating docking experiments, we made sure that the docking method would be efficient and produce reliable binding poses. To do so, the co-crystalized ligand was re-docked inside the M^pro^ active site. The resulting top-ranked pose was almost identical to that of the co-crystallized one with an RMSD of 1.1 Å (Figure S12).

The resulting binding poses of the three compounds were then visually investigated. All generated poses for the three compounds showed docking scores between −5.0 and −8.3 kcal/mol (Figure S13). Only poses with scores < −7 kcal/mol were selected for subsequent in silico investigation that was also convergent in their orientations inside the M^pro^ active site.

Second (Δ*G*_bind_ estimation step), these selected poses were subjected to molecular dynamic simulations (MDS)-based binding free energy estimation (Δ*G*_bind_) to select the best binding pose in terms of affinity towards M^pro^ active site. The Δ*G*_bind_ values of the best-ranked poses of compounds **1**–**3** were −8.1, −8.0, −3.2 kcal/mol, respectively. These results clearly explain why compound **3** displayed the least in vitro enzyme inhibitory activity. However, it is not sufficient to discriminate between **1** and **2** that were significantly different in their in vitro inhibitory activities against M^pro^ (IC_50_ = 0.47 and 3.9 µM, respectively).

**Figure 4:**
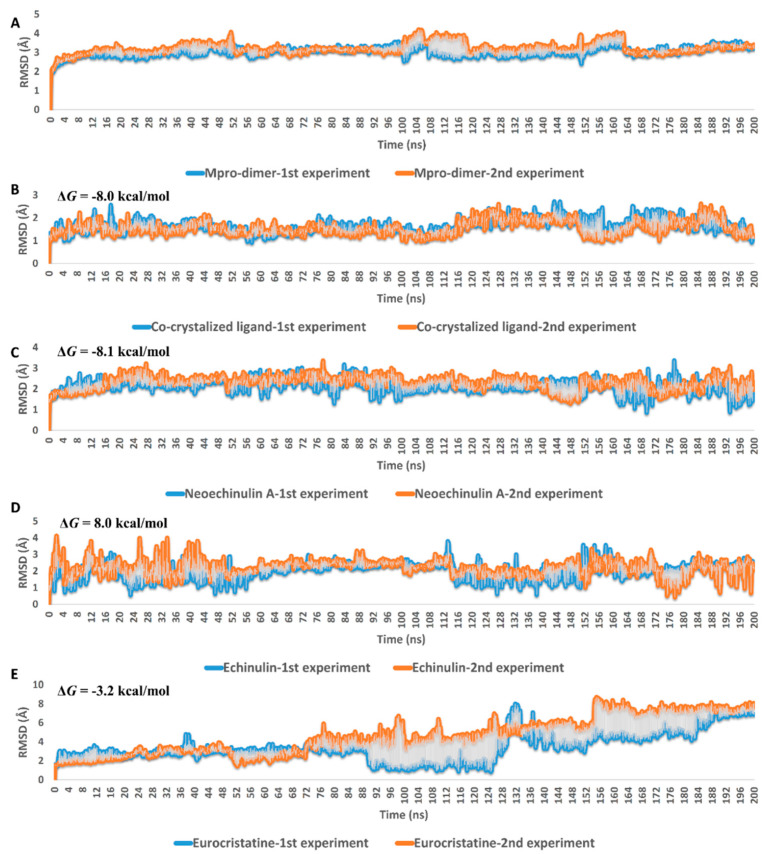
RMSDs of neoechinulin A (**1**), echinulin (**2**), and eurocristatine (**3**) (**C**–**E**) inside the M^pro^-dimer active site over the course of MDS. (**A**,**B**) are the RMSDs of the free dimeric form of M^pro^ and co-crystalized inhibitor over the course of MDS. The MDS experiments were carried out two times for 200 ns. The blue color indicates the 1st experiment, while the orange color indicates the 2nd one.

In the third step (MDS step), the binding pose of each compound was subjected to long MDS experiments carried out in duplicates to efficiently evaluate their stability inside the enzyme active site and investigate their mode of interaction. As shown in Figure 4, eurocristatine (**3**) was the least stable structure inside the M^pro^ active site, where it started to significantly deviate from the starting binding pose to reach RMSD > 7 Å at the end of the MDS. Regarding neoechinulin A (**1**) and echinulin (**2**), they both achieved stable binding inside the M^pro^ active site with average RMSDs of 2.16 Å and 2.21 Å, respectively. The dynamics of these two related structures (i.e., compounds **1** and **2**) over the course of the simulation were also convergent to that of the reported co-crystallized ligand (RMSD ~2 Å) [25] (Figure 4B).

**Figure 5:**
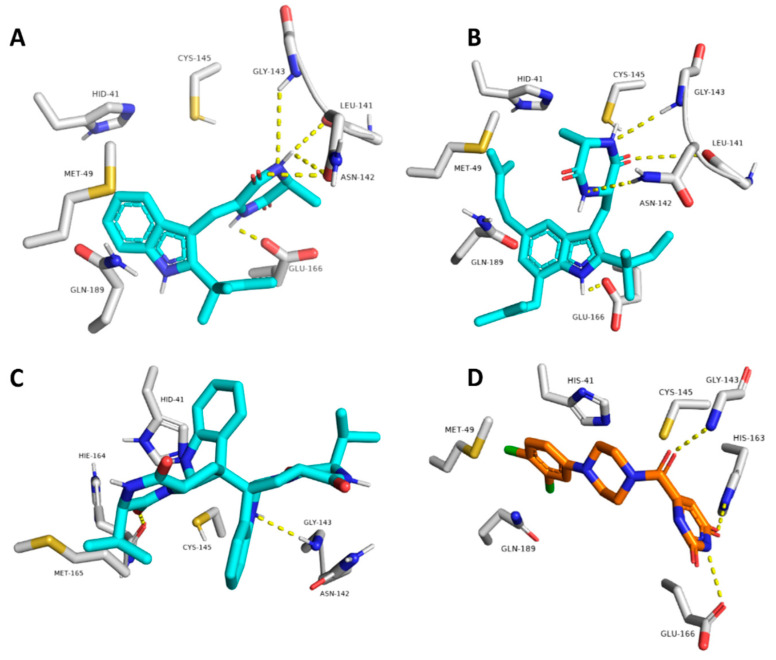
The most populated binding poses of neoechinulin A (**1**), echinulin (**2**), and eurocristatine (**3**) (cyan color) in addition to the co-crystalized inhibitor YD1 (orange color) (**A**–**D**, respectively) inside the M^pro^-dimer active site over the course of MDS.

**Figure 6:**
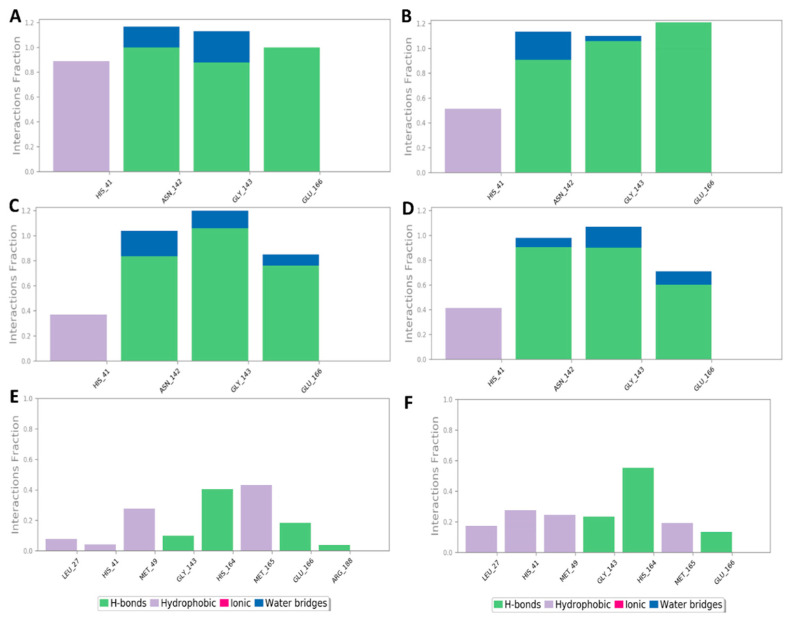
Protein–ligand contacts inside the M^pro^ active sites over 200 ns of MDS: (**A**,**B**) M^pro^-neoechinulin A (**1**) contacts derived from two independent MDS experiments, (**C**,**D**) M^pro^-echinulin (**2**) contacts derived from two independent MDS experiments, and (**E**,**F**) M^pro^-eurocristatine (**3**) contacts derived from two independent MDS experiments.

To investigate the hotspot amino acid residues that contributed to the binding stability of each compound, including the co-crystalized inhibitor (i.e., YD1), the most populated poses of each simulated ligand were extracted (Figure 5). Additionally, the ligand–protein interaction pattern was extracted during the whole MDS runs (Figure 6). As revealed in Figure 5 and Figure 6, neoechinulin A (**1**) and echinulin (**2**) binding poses populated 35% and 29% over their MDS, and both revealed stable H-bonds between the two compounds and LEU-141, ASN-142, GLY-143, and GLU-166 in addition to significant hydrophobic interaction with HIS-41. The main difference between the interactions of both compounds was that neoechinulin A (**1**) established the four H-bonds via its diketopiperidine moiety, while echinulin (**2**) established three H-bonds (i.e., with LEU-141, ASN-142, GLY-143) via its diketopiperidine moiety and the last H-bond with GLU-166 was established via its indole NH. Water bridges with ASN-142, GLY-143, and GLU-166 were also established, however, they showed low contribution in the binding stability of both compounds (Figure 4 and Figure 5).

The co-crystallized inhibitor YD1 also established two strong H-bonds with GLY-143 and GLU-166 as did both compounds **1** and **2**, in addition to further H-bond with HIS-163. Moreover, it also formed stable hydrophobic interactions with HIS-41 as in both compounds.

In regard to eurocristatine (**3**), its most populated pose (11%) revealed transient H-bonds with ASN-142, HIS-164, and GLU-166 in addition to a number of unstable hydrophobic interactions with LEU-27, HIS-41, MET-49, MET-165 (Figure 4 and Figure 5).

**Figure 7:**
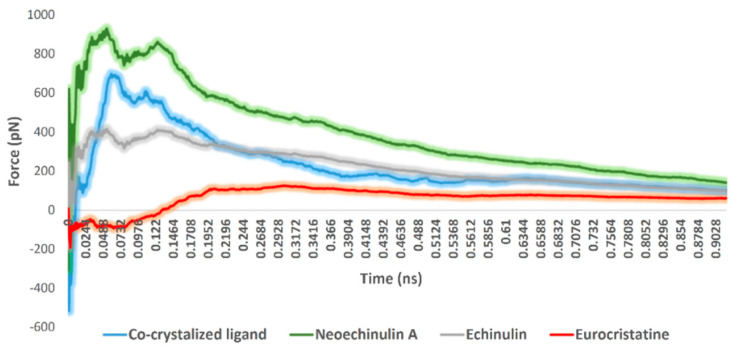
Comparison of the unbinding force profiles of neoechinulin A (**1**), echinulin (**2**), and eurocristatine (**3**) along with the co-crystalized ligand YD1. The plots show the resulting mean values from averaging the force profiles from three different SMD runs.

The fourth step, steered molecular dynamics (SMD), was carried out to determine the relative binding affinity of the investigated compounds, particularly neoechinulin A (**1**) and echinulin (**2**), that the calculated Δ*G*_bind_ were too convergent (−8.1 and −8.0 kcal/mol, respectively). However, **1** was eight times more active than **2** towards M^pro^ in vitro (Figure 3). For each, compound-protein complex (i.e., docking pose) and external force were applied to the compound (i.e., ligand) to steer it out of the M^pro^ active site. The exerted forces could be monitored over the course of the simulation. As shown in Figure 7, the unbinding of eurocristatine (**3**) (i.e., the inactive compound) was achieved by a force not higher than ~108 pN, while active compounds neoechinulin A (**1**) and echinulin (**2**) along with the co-crystalized inhibitor YD1 required an unbinding force ranging from 399 pN to 909 pN. The force profile plotted in Figure 7 clearly indicated that neoechinulin A (**1**) (IC_50_ = 0.47 µM) has the highest binding stability inside the M^pro^ active site (i.e., needed unbinding force ~909 pN), followed by the co-crystalized inhibitor YD1 (*K*i = 2.9 µM) [25] (i.e., needed unbinding force ~685 pN) followed by echinulin (**2**) (IC_50_ = 3.9 µM) (i.e., needed unbinding force ~399 pN). These differences in the unbinding forces between neoechinulin A (**1**), echinulin (**2**), and the co-crystalized inhibitor YD1 may be attributed to the degree of ligand–protein H-bonds’ stability during the unbinding process in addition to the degree of hydrophobic interactions with hydrophobic residues inside the enzyme active site, particularly with HIS-41, where neoechinulin A (**1**) was able to establish a significant stable hydrophobic interaction with this hydrophobic residue (Figure 6A). Accordingly, these SMD-based findings clearly explain the significant difference in the activity of neoechinulin A (**1**) in comparison with echinulin (**2**) depending on revealing how tightly each ligand was bound to the enzyme’s active site.

## 3. Discussion

The unprecedented pandemic of SARS-CoV-2 emerged in late 2019 is associated with significant mortality globally. Although several vaccines were developed, drug discovery campaigns are still crucial as vaccines alone were not sufficient for complete protection against the virus and its emerging variants. The SARS-CoV-2 M^pro^ is considered a promising drug target, as it is dissimilar to human proteases [26]. SARS-CoV-2: M^pro^ is a key enzyme in coronaviruses as it possesses an essential role in mediating viral replication and transcription. This made it attractive as a drug target for SARS-CoV-2, which has been used through different computer-assisted programs to discover medications that could modulate it and hence show good antiviral potential [3,27].

Although the computer-aided drug discovery process is very promising during pandemics, the standard bioguided isolation approach can quickly discover active hits. The marine fungal isolate *Aspergillus fumigatus* MR2012 was recovered from the Red Sea in 2011 and proved to be one of the “talented” strains in terms of drug discovery. Its initial large-scale fermentation on malt extract sea salt medium led to the isolation of nine diketopiperazine alkaloids that showed antibacterial activity against Gram-positive bacteria [28]. When *Aspergillus fumigatus* MR2012 was co-cultured with the hyper-arid desert bacterial isolate *Streptomyces leeuwenhoekii* strain C34 on ISP2 medium, it produced the two new metabolites luteoride D and pseurotin G. Using OSMAC approach and screening the strain against three different media led to the isolation of the new metabolite brevianamide X [29].

Herein, bioguided screening to discover potential SARS-CoV-2 M^pro^ inhibitors led to the selection of *Aspergillus fumigatus* MR2012 as its extract on a medium supplemented with mannitol, and showed good inhibition of SARS-CoV-2 M^pro^ in vitro. The large-scale fermentation of the fungus on this medium led to the isolation of three indole diketopiperazines and one isocoumarin derivative which were not traced before in the previous fungal fermentation studies. In vitro screening of the isolated metabolites highlighted that neoechinulin A (**1**) had significant SARS-CoV-2 M^pro^ inhibitory effects with IC_50_ value comparable to the positive control GC376. Neoechinulin A (**1**) has previously shown diverse biological effects such as antioxidant [30], neuroprotective [31], anti-inflammatory [32], and memory enhancer [33]. Although neoechinulin A has never been reported with antiviral effect, its closely related analogue neoechinulin B displayed potent inhibition against a panel of drug-resistant influenza clinical isolates by targeting the influenza envelope haemagglutinin and disrupting its interaction with the host cells [34].

Molecular modeling and simulation approaches became able to afford a very good explanation about the ligands’ mode of actions at the molecular level. After predicting the binding modes of neoechinulin A (**1**), echinulin (**2**), and eurocristatine (**3**) inside the M^pro^ active site via running multiple docking and Δ*G*_bind_ calculation trials, we initiated a number of MDS-based experiments to explain the reasons behind eurocristatine (**3**) being inactive and why neoechinulin A (**1**) was significantly more active than echinulin (**2**).

MDS-derived findings clearly showed that eurocristatine (**3**) had a low affinity toward M^pro^ active site. This conclusion came from (i) its significantly low calculated Δ*G*_bind_ value, (ii) its high average RMSD over the MDS runs, (iii) and its low calculated unbinding force. Additionally, the extracted binding behavior revealed its transient molecular interactions inside the enzyme’s active site.

Accordingly, the second question about the difference in the in vitro activity between the structurally related neoechinulin A (**1**) and echinulin (**2**) was explained by calculating their relative binding stabilities inside the M^pro^ active site by estimating the force needed to pull each compound out of the active site via running a number of SMD experiments. To the best of our knowledge, this is the first non-classical MDS-based experiment (i.e., SMD) in the relative affinity estimation of potential SARS CoV-2 M^pro^ inhibitors.

Depending on the previous discussion, it is important to emphasize the following concluding points that should be taken into consideration in dealing with the M^pro^-related in silico investigation: (i) long multiple classical MDS runs (>150 ns long) produce sufficient data regarding the binding stability of the proposed ligands inside the M^pro^’s active site. Shorter single runs (<50 ns) might be misleading on some occasions and unable to differentiate between active and inactive ligands [35]; (ii) MDS experiments should be carried out using the M^pro^ dimeric model to get more accurate and realistic results [36]; (iii) absolute binding free energy estimation and MDS experiments in some cases are not sufficient to discriminate between structurally similar scaffolds. Hence, using different non-classical MDS experiments could be the best solution in such cases [37].

It is worth noting that both neoechinulin A (**1**) and echinulin (**2**) have good drug-like properties according to Lipinski’s rules [38] in contrast to that of eurocristatine (**3**) which has poor drug-like properties. Hence, these diketopiperazine derivatives, particularly neoechinulin A (**1**), are considered a very promising non-covalent M^pro^ inhibitors that can be developed as natural product-based COVID-19 medication.

## 4. Materials and Methods

### 4.1. Chemicals

All solvents used for extraction and processing were purchased from Fisher Chemical UK while HPLC grade solvents were obtained from Rathburn Chemicals Ltd. (Walkerburn, UK). Column chromatography was performed on Acros Organics 0.03–0.20 mm mesh size silica gel. Thin layer chromatography (TLC) technique was used for preliminary identification and performed on pre-coated TLC sheets POLYGRAM^®^ SIL G/UV_254_, Macherey-Nagel GmbH & Co., and samples were spotted using 5 μL micropipettes (Drummond Scientific Company, Broomall, PA, USA). Silica plates were visualized under UVGL Handheld UV lamp and para-Anisaldehyde (99% pure, Acros Organics, 2440 Geel, Belgium). Deuterated DMSO (Cambridge Isotope Laboratories, Inc., Tewksbury, MA, USA) was used for NMR measurements. The ingredients used to prepare fungal culture media were purchased from Oxiod, Hampshire RG24 8PW, UK.

### 4.2. Instruments

For compound purification, Agilent 1100 series HPLC system connected to Diode Array Detector was used. HRESIMS data were obtained using a Thermo LTQ XL/LTQ Orbitrap Discovery MS system coupled with an HPLC system (PDA detector, PDA autosampler and pump) run under the following conditions: capillary voltage of 45 V, capillary temperature of 260 °C, auxiliary gas flow rate of 10–20 arbitrary units, sheath gas flow rate of 40–50 arbitrary units, spray voltage of 4.5 kV, and mass range of 100–2000 amu (maximal resolution of 30,000). A Sunfire C_18_ analytical HPLC column (5 μm 4.6 mm × 150 mm) was employed with a mobile phase of 0 to 100% MeOH over 30 min at a flow rate of 1 mL/min for the liquid chromatography analysis. Structure characterization of compounds was based on 1D and 2D NMR data, using Bruker Advance III spectrometer 600 MHz (Bruker UK Ltd., Durham, UK).

### 4.3. Fungal Isolation and Identification

The fungal isolate MR2012 was recovered from the marine sediment collected from Hurgada, the Red Sea, at the end of 2011. Sequence analysis of the ITS regions of rDNA showed 99% identity with *Aspergillus fumigatus* [28].

### 4.4. Small- and Large-Scale Fermentation and Extraction

The pure fungal culture was grown in ISP2 agar media (supplemented with 25 g/L sea salt) and incubated at room temperature for 5–7 days. Approximately 5 × 5 mm size agar pieces with the fungus were aseptically introduced into ten 3 L conical flasks containing 800 mL of fermentation broth containing 10 g/L of malt extract, 10 g/L of yeast extract, 4 g/L of mannitol, and supplemented with 25 g/L sea salt, under static fermentation for 28 days at room temperature. Then, the mycelial bed in each flask was combined and extracted by sonication with methanol (MeOH) (3 × 500 mL) for 1 h each. The MeOH extract was evaporated under vacuum to get 3.8 g of the crude extract. This extract was resuspended in 200 mL of 1:1 distilled water and MeOH and subsequently fractionated with 3 × 250 mL of *n*—hexane, DCM, and EtOAc, which were also evaporated under vacuum. TLC analysis confirmed different patterns in all fractions.

### 4.5. Isolation of Fungal Metabolites

The biologically active DCM fraction (1.1 g) was further purified through its injection into RP–HPLC (Phenomenex RP–C_18_ analytical column (Luna 5 μm, 250 × 4.60 mm, L × i.d.)) using a gradient of 25–100% of MeCN in H_2_O for 30 min and 100% MeCN for 5 min at a flow rate of 2 mL/min. This method afforded the four major compounds 1 (4.2 mg), 2 (1.1 mg), 3 (2.4 mg), and 4 (2.7 mg).

Neoechinulin A (**1**): white powder; [α]^25^_D_ − 66.65 (c 0.15, CH_3_OH); UV (MeOH) λ_max_ (log ε): 225 (3.95), 288 (3.15), 326 (2.90); ^1^H (600 MHz, DMSO–*d*_6_): *δ* 1.38 (3H, d, J = 6.5), 1.48 (6H, s), 5.05 (2H, s), 6.08 (1H, m), 2.55 (2H, s), 8.67 (1H, s), 4.17 (1H, d, J = 7.0), 6.89 (1H, s), 7.42 (1H, d, J = 8.0), 7.01 (1H, t, J = 7.0, 7.8), 7.09 (1H, t, J = 8.0, 7.0), 7.19 (1H, d, J = 8), 11.06 (1H, s); ^13^C NMR (150 MHz, DMSO–*d*_6_): 20.0 (C-30), 28.0 (C-18/19), 112.1 (C-17), 145.6 (C-16), 40.9 (C-15), 166.9 (C-13), 51.0 (C-12), 160.4 (C-10), 125.3 (C-9), 110.7 (C-8), 135.6 (C-7a), 112.0 (C-7), 119.8 (C-6), 121.2 (C-5), 119.4 (C-4), 126.5 (C-3a), 103.9 (C-3), 144.4 (C-2); HR–ESIMS: [M + H]^+^ at *m*/*z* 324.1719 C_19_H_22_N_3_O_2_ (calcd. 324.1707).

Echinulin (**2**): yellow powder; [α]^25^_D_ − 26.15 (c 0.2, CH_3_OH); UV (MeOH) λ_max_ (log ε): 218 (4.10), 284 (2.95); ^1^H (600 MHz, DMSO–*d*_6_): *δ* 1.33 (3H, d, J = 7), 1.69 (6H, s), 1.74 (6H, s), 5.38 (1H, t, J = 7.0, 7.5), 3.56 (2H, d, J = 7.6), 1.69 (6H, s), 1.74 (6H, s), 5.30 (1H, t, J = 7.0, 7.5), 3.29 (2H, d, J = 6.7), 1.50 (6H, d, J = 7.4), 1.50 (6H, d, J = 7.4), 5.05 (2H, m), 6.21 (1H, dd, J = 17.5, 10.6), 8.17 (1H, s), 3.81 (1H, dd, J = 2.8, 3.1), 7.43 (1H, s), 3.91 (1H, m), 3.01 (2H, dd, J = 9.4, 10), 6.63 (1H, s), 7.04 (1H, s), 9.70 (1H, s); ^13^C NMR (150 MHz, DMSO–*d*_6_): 21.3 (C-30), 18.2 (C-28/29), 130.8 (C-27), 123.3 (C-26), 29.5 (C-25), 26.0 (C-23/24), 132.0 (C-22), 125.3 (C-21), 34.6 (C-20), 28.5 (C-18/19), 111.5 (C-17), 147.3 (C-16), 40.9 (C-15), 168.4 (C-13), 50.8 (C-12), 167.9 (C-10), 56.1 (C-9), 31.8 (C-8), 141.9 (C-7a), 132.7 (C-7), 121.4 (C-6), 124.0 (C-5), 115.1 (C-4), 129.7 (C-3a), 105.6 (C-3), 145.9 (C-2); HR–ESIMS: [M + H]^+^ at *m*/*z* 462.3119 C_29_H_40_N_3_O_2_ (calcd. 462.3115).

Eurocristatine (**3**): white powder; [α]^25^_D_ + 295.50 (c 0.02, CH_3_OH); UV (MeOH) λ_max_ (log ε): 212 (4.15), 244 (3.95), 304 (3.60); ^1^H (600 MHz, DMSO–*d*_6_): *δ* 0.69 (3H, d, J = 7), 0.81 (3H, d, J = 7), 1.95 (1H, m), 3.39 (1H, t, J = 5, 4.9), 8.26 (1H, d, J = 4.3), 3.14 (2H, s), 2.39 (2H, m), 4.12 (1H, t, J = 8.8, 8.8), 6.60 (1H, d, J = 7.7), 7.03 (1H, t, J = 7.5, 7.7), 6.64 (1H, t, J = 7.5, 7.5), 7.40 (1H, d, J = 7.5), 4.90 (1H, s), 6.74 (1H, s); ^13^C NMR (150 MHz, DMSO–*d*_6_): *δ* 18.5 (C-19), 19.5 (C-18), 32.5 (C-17), 167.9 (C-16), 62.8 (C-15), 168.8 (C-13), 37.7 (C-12α), 56.2 (C-11), 149.6 (C-9), 109.4 (C-8), 129.2 (C-7), 118.4 (C-6), 124.9 (C-5), 130.9 (C-4), 60.2 (C-3), 79.5 (C-2); HR–ESIMS: [M + H]^+^ at *m*/*z* 569.2871 C_32_H_37_N_6_O_4_ (calcd. 569.2871).

Eurotiumide G (**4**): yellow oil; [α]^25^_D_ 0 (c 0.10, CH_3_OH, suggesting a racemic mixture); UV (MeOH) λ_max_ (log ε): 220 (3.25), 263 (0.90), 335 (1.2); ^1^H (600 MHz, DMSO–*d*_6_): *δ* 9.13 (1H, d, J = 2.2), 3.31 (3H, s), 3.38 (3H, br s), 1.30 (3H, br s), 1.30 (3H, br s), 5.75 (1H, d, J = 9.5), 6.32 (1H, d, J = 9.7), 0.89 (3H, t, J = 6.7, 6.6), 1.33 (6H, s), 1.32 (6H, s), 1.50 (2H, m), 1.39 (2H, m), 1.64 (2H, m), 1.70 (2H, m), 6.53 (1H, s), 4.11 (1H, d, J = 2), 3.87 (1H, br s), 5.35 (1H, s); ^13^C NMR (150 MHz, DMSO–*d*_6_): *δ* 58.0 (4-OCH3), 55.6 (1-OCH3), 27.7 (C-5″), 27.5 (C-4″), 75.7 (C-3″), 132.6 (C-2″), 122.4 (C-1″), 14.6 (C-5′), 22.6 (C-4′), 31.7 (C-3′), 25.5 (C-2′), 30.7 (C-1′), 123.4 (C-8a), 142.3 (C-8), 121.5 (C-7), 112.4 (C-6), 149.2 (C-5), 117.8 (C-4a), 69.3 (C-4), 70.5 (C-3), 95.0 (C-1); HR–ESIMS: [M + H]^+^ at *m*/*z* 363.2160 C_21_H_31_O_5_ (calcd. 363.2166).

### 4.6. In Vitro Assay against SARS-CoV-2 Main Protease

The fungal extract, fractions, and the isolated compounds (**1**–**4**) were assessed for their in vitro enzyme inhibition activities using 3CL Protease, tagged (SARS-CoV-2) Assay Kit (Catalog #: 79955-1, BPS Bioscience, Inc., Allentown, PA, USA), according to manufacturer’s protocol [15]. The in vitro FRET assay was monitored at an emission wavelength of 460 nm with excitation at 360 nm, using a Flx800 fluorescence spectrophotometer (BioTek Instruments, Winooski, VT, USA).

### 4.7. Docking and Molecular Dynamics Simulations

The in silico-based investigation was carried out in four steps:

Step1 (docking step): the compounds under investigation were docked into the M^pro^ active site using Autodock Vina [39]. The resulting top-ranked pose was almost identical to that of the co-crystallized one with an RMSD of 1.1 Å (Figure S12). The generated binding poses were then visually investigated using Pymol software [13,39].
Step 2 (Δ*G*_bind_ estimation step): Top-scoring poses resulted from the docking step were subjected to molecular dynamic simulations (MDS)-based binding free energy estimation (Δ*G*_bind_) using the Free Energy Perturbation (FEP) method [40]. MDS experiments carried out to calculate Δ*G*_bind_ were performed by NAMD software [41], and all input files required for simulation by NAMD were prepared by using the online website Charmm-GUI (https://charmm-gui.org/?doc=input/afes.abinding, accessed on 15 February 2022) [40,41].
Step 3 (MDS step): Top-scoring poses resulted from the docking step were subjected to 200 ns MD simulations carried out in duplicates. MDS runs were performed and analyzed by Desmond software [14,15,42,43].
Step 4 (SMD step): steered molecular dynamics (SMD), was carried out to determine the relative binding affinity of the investigated compounds. All SMD experiments were carried out by NAMD software as described previously [41].

The detailed procedures for each step are described in the Supplementary file.

## 5. Conclusions

Herein, we have fermented our in-house marine fungal strains on different media using the OSMAC approach. Through bio-guided screening, we have discovered the indole diketopiperazine alkaloid neoechinulin A as a potential structural motif against SARS-CoV-2 M^pro^. By applying a number of different modeling and biophysics-based simulation experiments, the binding mode of this promising natural product along with its congeners was putatively elucidated. Additionally, we introduced SMD as a simple and powerful MDS-based method to discriminate between active and inactive structurally related motifs.

## Data Availability

Not applicable.

## Supplementary Materials

The following are available online at https://www.mdpi.com/article/10.3390/md20030163/s1, Figures S1–S14 including HRMS and NMR of the isolated metabolites and docking analysis. 15. Methods and References. References [44,45,46,47,48] are cited in the supplementary materials.

## References

[1] https://covid19.who.int/.

[2] https://news.un.org/en/story/2021/04/1089392.

[3] Jin Z., Du X., Xu Y., Deng Y., Liu M., Zhao Y., Zhang B., Li X., Zhang L., Peng C. (2020). Structure of M^pro^ from SARS-CoV-2 and discovery of its inhibitors. Nature.

[4] Ng Y.L., Salim C.K., Chu J.J.H. (2021). Drug repurposing for COVID-19: Approaches, challenges and promising candidates. Pharmacol. Ther..

[5] Mercorelli B., Palù G., Loregian A. (2018). Drug repurposing for viral infectious diseases: How far are we?. Trends Microbiol..

[6] https://www.fda.gov/consumers/consumer-updates/know-your-treatment-options-covid-19.

[7] https://www.ema.europa.eu/en/human-regulatory/overview/public-health-threats/coronavirus-disease-covid-19/treatments-vaccines/covid-19-treatments.

[8] Jayk Bernal A., Gomes da Silva M.M., Musungaie D.B., Kovalchuk E., Gonzalez A., Delos Reyes V., Martín-Quirós A., Caraco Y., Williams-Diaz A., Brown M.L. (2022). Molnupiravir for Oral Treatment of COVID-19 in Nonhospitalized Patients. N. Engl. J. Med..

[9] Owen D.R., Allerton C.M., Anderson A.S., Aschenbrenner L., Avery M., Berritt S., Boras B., Cardin R.D., Carlo A., Coffman K.J. (2021). An oral SARS-CoV-2 M^pro^ inhibitor clinical candidate for the treatment of COVID-19. Science.

[10] Mahase E. (2021). COVID-19: Pfizer’s paxlovid is 89% effective in patients at risk of serious illness, company reports. Br. Med. J..

[11] Newman D.J., Cragg G.M. (2020). Natural products as sources of new drugs over the nearly four decades from 01/1981 to 09/2019. J. Nat. Prod..

[12] Caly L., Druce J.D., Catton M.G., Jans D.A., Wagstaff K.M. (2020). The FDA-approved drug ivermectin inhibits the replication of SARS-CoV-2 in vitro. Antivir. Res..

[13] Thissera B., Sayed A.M., Hassan M.H.A., Abdelwahab S.F., Amaeze N., Semler V.T., Alenezi F.N., Yaseen M., Alhadrami H.A., Belbahri L. (2021). Bioguided Isolation of Cyclopenin Analogues as Potential SARS-CoV-2 M^pro^ Inhibitors from *Penicillium citrinum* TDPEF34. Biomolecules.

[14] Salih A.E., Thissera B., Yaseen M., Hassane A.S., El-Seedi H.R., Sayed A.M., Rateb M.E. (2021). Marine sulfated polysaccharides as promising antiviral agents: A comprehensive report and modeling study focusing on SARS CoV-2. Mar. Drugs.

[15] Alhadrami H.A., Sayed A.M., Al-Khatabi H., Alhakamy N.A., Rateb M.E. (2021). Scaffold Hopping of α-Rubromycin Enables Direct Access to FDA-Approved Cromoglicic Acid as a SARS-CoV-2 MPro Inhibitor. Pharmaceuticals.

[16] Alhadrami H.A., Sayed A.M., Hassan H.M., Youssif K.A., Gaber Y., Moatasim Y., Kutkat O., Mostafa A., Ali M.A., Rateb M.E. (2021). Cnicin as an Anti-SARS-CoV-2: An Integrated in Silico and in Vitro Approach for the Rapid Identification of Potential COVID-19 Therapeutics. Antibiotics.

[17] Alhadrami H.A., Sayed A.M., Sharif A.M., Azhar E.I., Rateb M.E. (2021). Olive-Derived Triterpenes Suppress SARS-CoV-2 Main Protease: A Promising Scaffold for Future Therapeutics. Molecules.

[18] Orfali R., Rateb M.E., Hassan H.M., Alonazi M., Gomaa M.R., Mahrous N., GabAllah M., Kandeil A., Perveen S., Abdelmohsen U.R. (2021). Sinapic Acid Suppresses SARS CoV-2 Replication by Targeting Its Envelope Protein. Antibiotics.

[19] Reher R., Kim H.W., Zhang C., Mao H.H., Wang M., Nothias L.F., Caraballo-Rodriguez A.M., Glukhov E., Teke B., Leao T. (2020). A convolutional neural network-based approach for the rapid annotation of molecularly diverse natural products. J. Am. Chem. Soc..

[20] van Santen J.A., Poynton E.F., Iskakova D., McMann E., Alsup T.A., Clark T.N., Fergusson C.H., Fewer D.P., Hughes A.H., McCadden C.A. (2021). The Natural Products Atlas 2.0: A database of microbially-derived natural products. Nucleic Acids Res..

[21] Wei X., Feng C., Wang S.Y., Zhang D.M., Li X.H., Zhang C.X. (2020). New Indole Diketopiperazine Alkaloids from Soft Coral-Associated Epiphytic Fungus Aspergillus sp. EGF 15-0-3. Chem. Biodivers..

[22] Zou X., Li Y., Zhang X., Li Q., Liu X., Huang Y., Tang T., Zheng S., Wang W., Tang J. (2014). A new prenylated indole diketopiperazine alkaloid from *Eurotium cristatum*. Molecules.

[23] Gomes N.M., Dethoup T., Singburaudom N., Gales L., Silva A.M., Kijjoa A. (2012). Eurocristatine, a new diketopiperazine dimer from the marine sponge-associated fungus *Eurotium cristatum*. Phytochem. Lett..

[24] Chen M., Shao C.L., Wang K.L., Xu Y., She Z.G., Wang C.Y. (2014). Dihydroisocoumarin derivatives with antifouling activities from a gorgonian derived *Eurotium* sp. fungus. Tetrahedron.

[25] Clyde A., Galanie S., Kneller D.W., Ma H., Babuji Y., Blaiszik B., Brace A., Brettin T., Chard K., Chard R. (2022). High Throughput Virtual Screening and Validation of a SARS-CoV-2 Main Protease Non-Covalent Inhibitor. J. Chem. Inf. Model..

[26] Ullrich S., Nitsche C. (2020). The SARS-CoV-2 main protease as drug target. Bioorganic Med. Chem. Lett..

[27] Zhu N., Zhang D., Wang W., Li X., Yang B., Song J., Zhao X., Huang B., Shi W., Lu R. (2020). A novel coronavirus from patients with pneumonia in China, 2019. N. Engl. J. Med..

[28] El-Gendy B.E.D.M., Rateb M.E. (2015). Antibacterial activity of diketopiperazines isolated from a marine fungus using t-butoxycarbonyl group as a simple tool for purification. Bioorg. Med. Chem. Lett..

[29] Wakefield J., Hassan H.M., Jaspars M., Ebel R., Rateb M.E. (2017). Dual induction of new microbial secondary metabolites by fungal bacterial co-cultivation. Front. Microbiol..

[30] Yagi R., Doi M. (1999). Isolation of an antioxidative substance produced by *Aspergillus repens*. Biosci. Biotechnol. Biochem..

[31] Kamisuki S., Himeno N., Tsurukawa Y., Kusayanagi T., Takeno M., Kamakura T., Kuramochi K., Sugawara F. (2018). Identification of proteins that bind to the neuroprotective agent neoechinulin A. Biosci. Biotechnol. Biochem..

[32] Kim K.S., Cui X., Lee D.S., Sohn J.H., Yim J.H., Kim Y.C., Oh H. (2013). Anti-inflammatory effect of neoechinulin a from the marine fungus *Eurotium* sp. SF-5989 through the suppression of NF-кB and p38 MAPK pathways in lipopolysaccharide-stimulated RAW264. 7 macrophages. Molecules.

[33] Sasaki-Hamada S., Hoshi M., Niwa Y., Ueda Y., Kokaji A., Kamisuki S., Kuramochi K., Sugawara F., Oka J.I. (2016). Neoechinulin A induced memory improvements and antidepressant-like effects in mice. Prog. Neuropsychopharmacol. Biol. Psychiatry.

[34] Chen X., Si L., Liu D., Proksch P., Zhang L., Zhou D., Lin W. (2015). Neoechinulin B and its analogues as potential entry inhibitors of influenza viruses, targeting viral hemagglutinin. Eur. J. Med. Chem..

[35] Muratov E.N., Amaro R., Andrade C.H., Brown N., Ekins S., Fourches D., Tropsha A. (2021). A critical overview of computational approaches employed for COVID-19 drug discovery. Chem. Soc. Rev..

[36] Komatsu T.S., Okimoto N., Koyama Y.M., Hirano Y., Morimoto G., Ohno Y., Taiji M. (2020). Drug binding dynamics of the dimeric SARS-CoV-2 main protease, determined by molecular dynamics simulation. Sci. Rep..

[37] Padhi A.K., Rath S.L., Tripathi T. (2021). Accelerating COVID-19 research using molecular dynamics simulation. J. Phys. Chem. B.

[38] Daina A., Michielin O., Zoete V. (2017). SwissADME: A free web tool to evaluate pharmacokinetics, drug-likeness and medicinal chemistry friendliness of small molecules. Sci. Rep..

[39] Seeliger D., de Groot B.L. (2010). Ligand docking and binding site analysis with PyMOL and Autodock/Vina. J. Comput. Aided Mol. Des..

[40] Kim S., Oshima H., Zhang H., Kern N.R., Re S., Lee J., Rous B., Sugita Y., Jiang W., Im W. (2020). CHARMM-GUI free energy calculator for absolute and relative ligand solvation and binding free energy simulations. J. Chem. Theory Comput..

[41] Phillips J.C., Braun R., Wang W., Gumbart J., Tajkhorshid E., Villa E., Chipot C., Skeel R.D., Kalé L., Schulten K. (2005). Scalable molecular dynamics with NAMD. J. Comput. Chem..

[42] Release S. (2017). 3: Desmond molecular dynamics system, DE shaw research, New York, NY, 2017. Maestro-Desmond Interoperability Tools.

[43] Schrodinger, LLC (2009). Maestro, Version 9.0.

[44] Sayed A.M., Alhadrami H.A., El-Gendy A.O., Shamikh Y.I., Belbahri L., Hassan H.M., Abdelmohsen U.R., Rateb M.E. (2020). Microbial natural products as potential inhibitors of SARS-CoV-2 main protease (Mpro). Microorganisms.

[45] Amaro R.E., Baudry J., Chodera J., Demir Ö., McCammon J.A., Miao Y., Smith J.C. (2018). Ensemble docking in drug discovery. Biophys. J..

[46] Bowers K.J., Chow D.E., Xu H., Dror R.O., Eastwood M.P., Gregersen B.A., Klepeis J.L., Kolossvary I., Moraes M.A., Sacerdoti F.D. (2006). Scalable algorithms for molecular dynamics simulations on commodity clusters. Proceedings of the SC’06: Proceedings of the 2006 ACM/IEEE Conference on Supercomputing.

[47] Ngo S.T., Tam N.M., Quan P.M., Nguyen T.H. (2021). Benchmark of Popular Free Energy Approaches Revealing the Inhibitors Binding to SARS-CoV-2 Mpro. J. Chem. Inf. Model..

[48] Tutone M., Perricone U., Almerico A.M. (2017). Conf-VLKA: A structure-based revisitation of the Virtual Lock-and-key Approach. J. Mol. Graph. Model..

