# Supplementary material for: Neoechinulin A as a Promising SARS-CoV-2 Mpro Inhibitor: In Vitro and In Silico Study Showing the Ability of Simulations in Discerning Active from Inactive Enzyme Inhibitors
Source: Mar Drugs. 2022 Feb 24;20(3):163. doi: 10.3390/md20030163 (PMC8955780; doi:10.3390/md20030163)
Supplement: Supplementary file 1 [file marinedrugs-20-00163-s001.zip › marinedrugs-1594708-supplementary.pdf]

Supplementary Information

Neoechinulin A as a promising SARS-CoV-2 M^{pro} inhibitor: in vitro and in silico study showing the ability of simulations in discerning active from inactive enzyme inhibitors

Hani Alhadrami [1,2,3,+], Gaia Burgio [4,+], Bathini Thissera [4], Raha Orfali [5], Suzan E. Jiffri [6], Mohammed Yaseen [4], Ahmed M. Sayed [7,*], and Mostafa E. Rateb [4,*]

Table of Content:

1. Figure S1 HRESIMS of compound **1**
2. Figure S2 ^{1}H NMR data of compound **1**
3. Figure S3 ^{13}C NMR data of compound **1**
4. Figure S4 HRESIMS of compound **2**
5. Figure S5 ^{1}H NMR data of compound **2**
6. Figure S6 ^{13}C NMR data of compound **2**
7. Figure S7 HRESIMS of compound **3**
8. Figure S8 ^{1}H NMR data of compound **3**
9. Figure S9 ^{13}C NMR data of compound **3**
10. Figure S10 HRESIMS of compound **4**
11. Figure S11 ^{1}H NMR data of compound **4**
12. Figure S12 ^{13}C NMR data of compound **4**
13. Figure S13 Binding modes of the co-crystalized ligand YD1 in its original and docking states inside the M^{pro} active site.
14. Figure S14 Different docking poses generated from the docking step of compounds (**1**–**3**)
15. Methods and references.

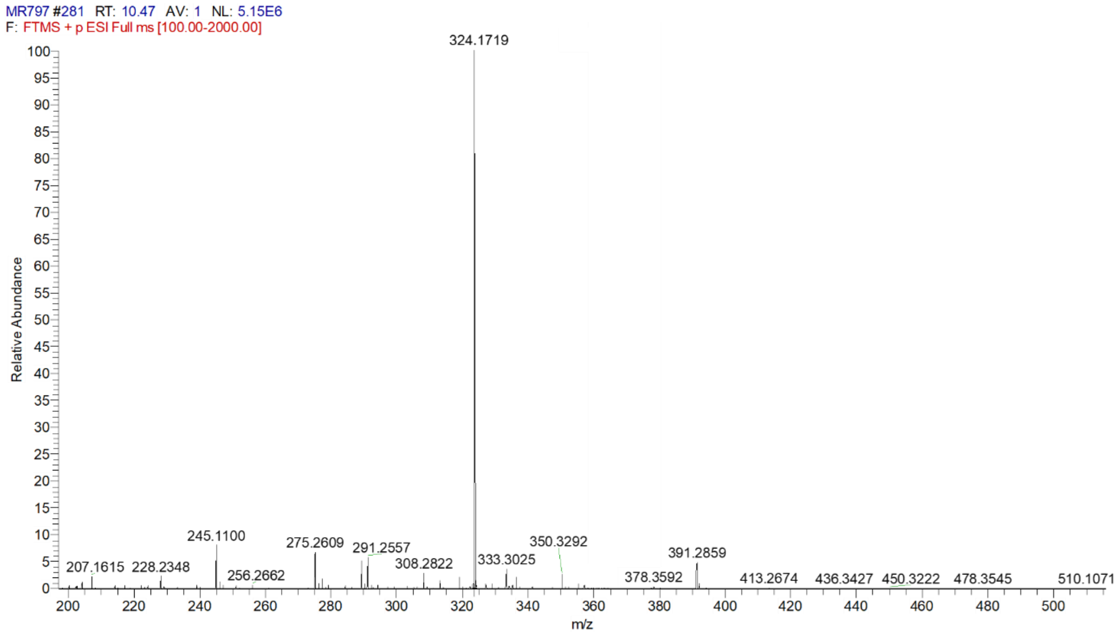

Figure S1 HRESIMS spectrum of compound **1**.

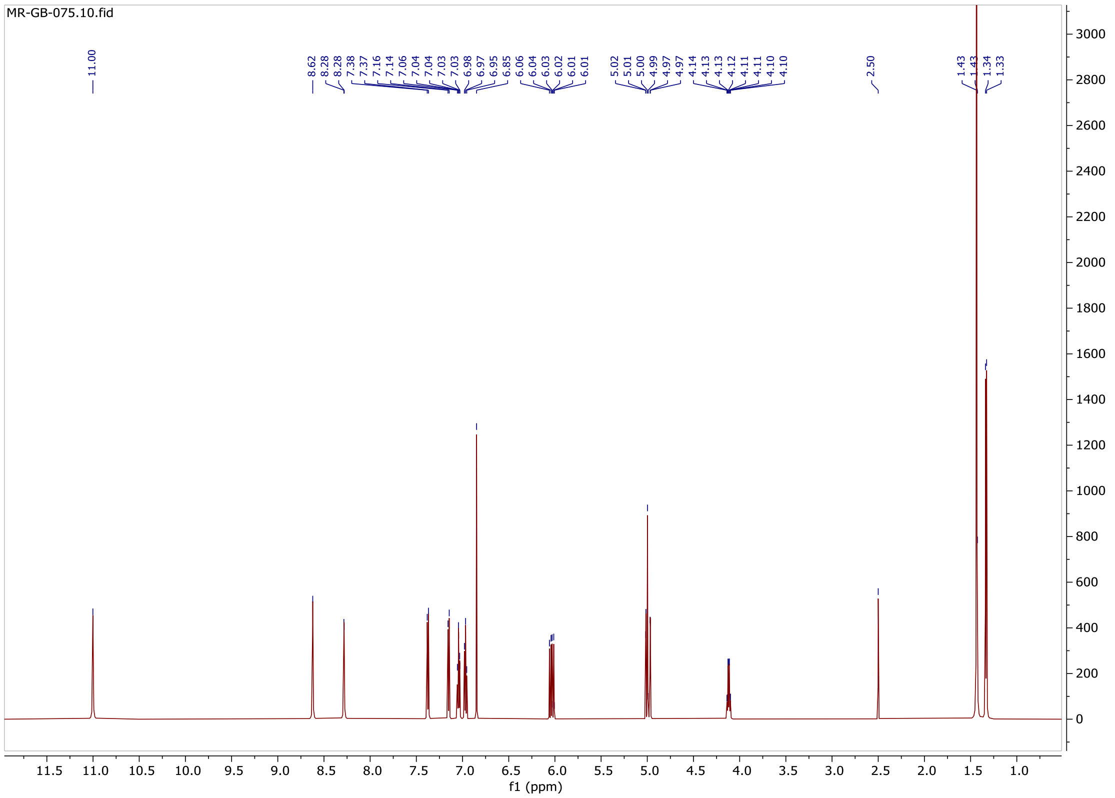

Figure S2 ^{1}H NMR spectrum of compound **1**.

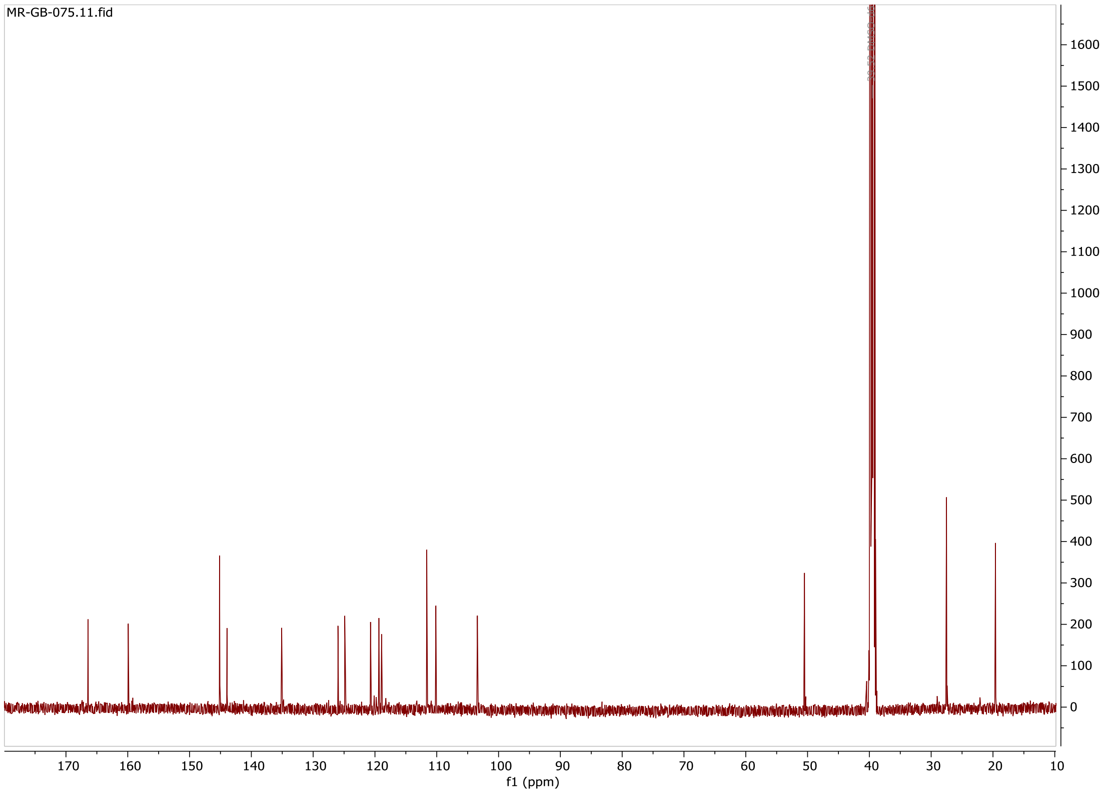

Figure S3 ^{13}C NMR spectrum of compound **1**.

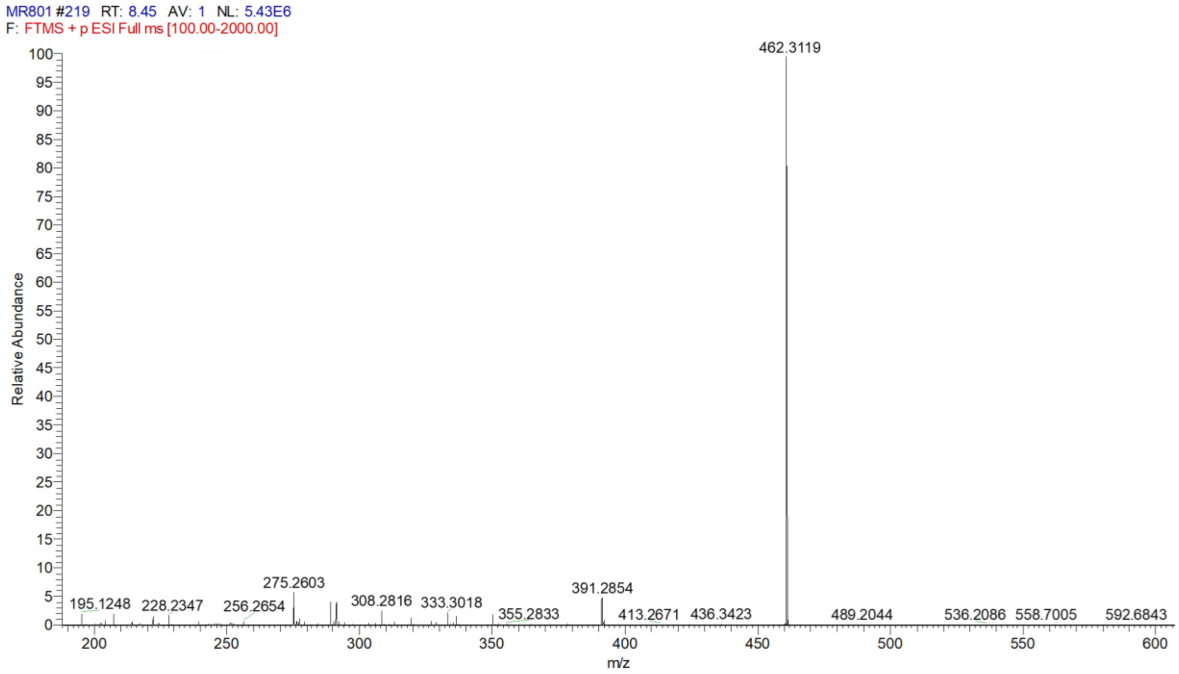

Figure S4 HRESIMS spectrum of compound **2**.

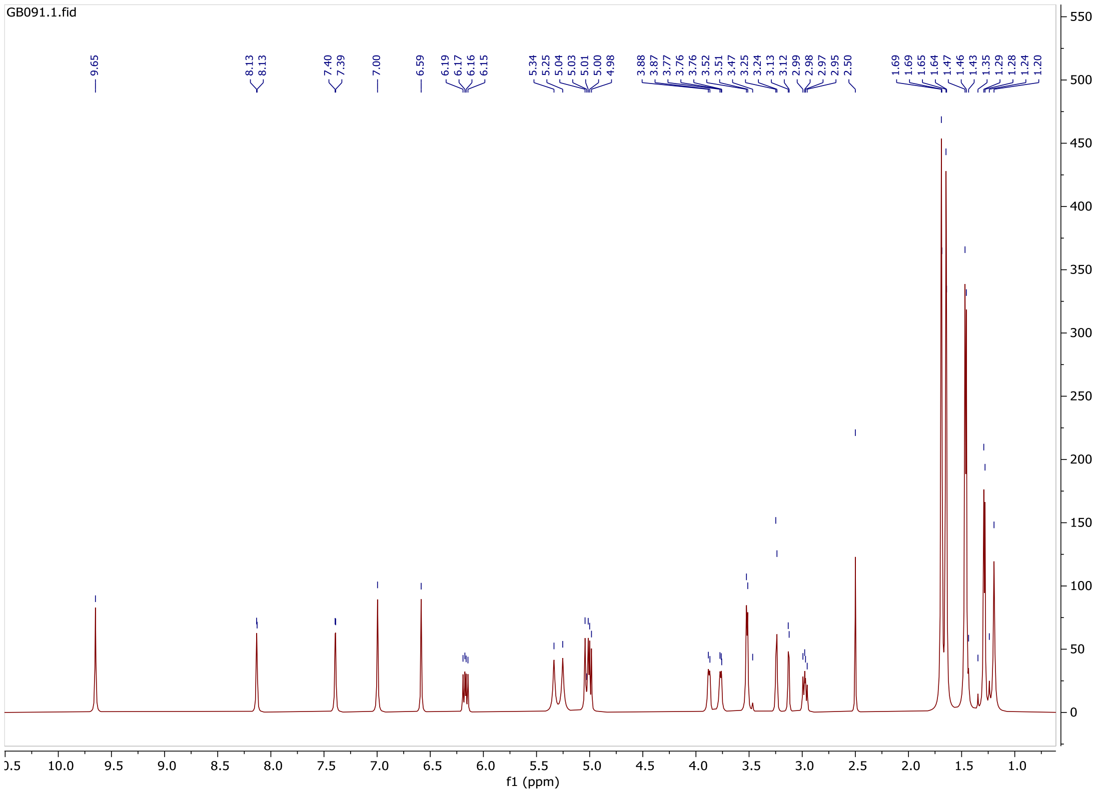

Figure S5 ^{1}H NMR spectrum of compound **2**.

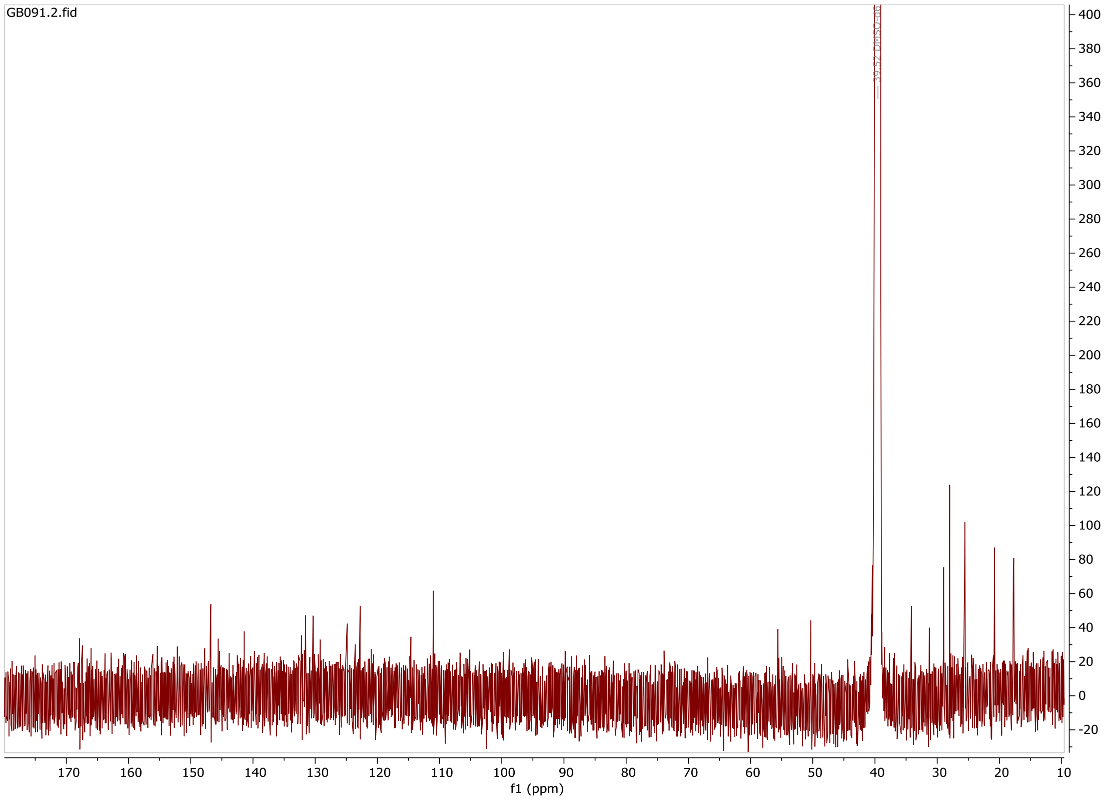

Figure S6 ^{13}C NMR spectrum of compound **2**.

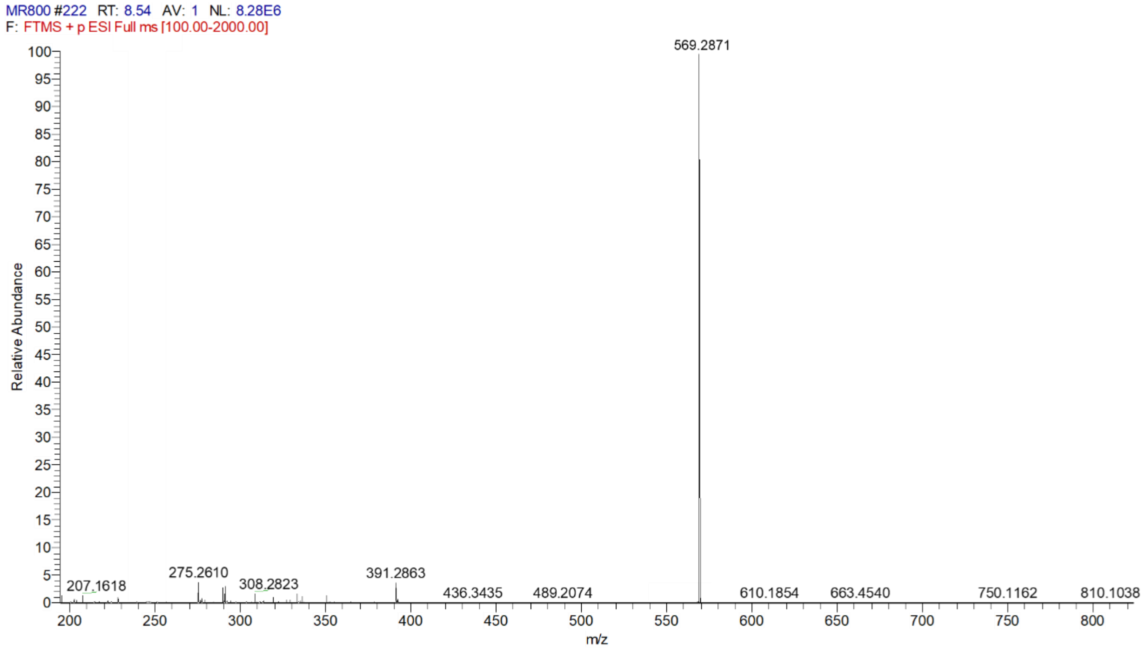

Figure S7 HRESIMS spectrum of compound **3**.

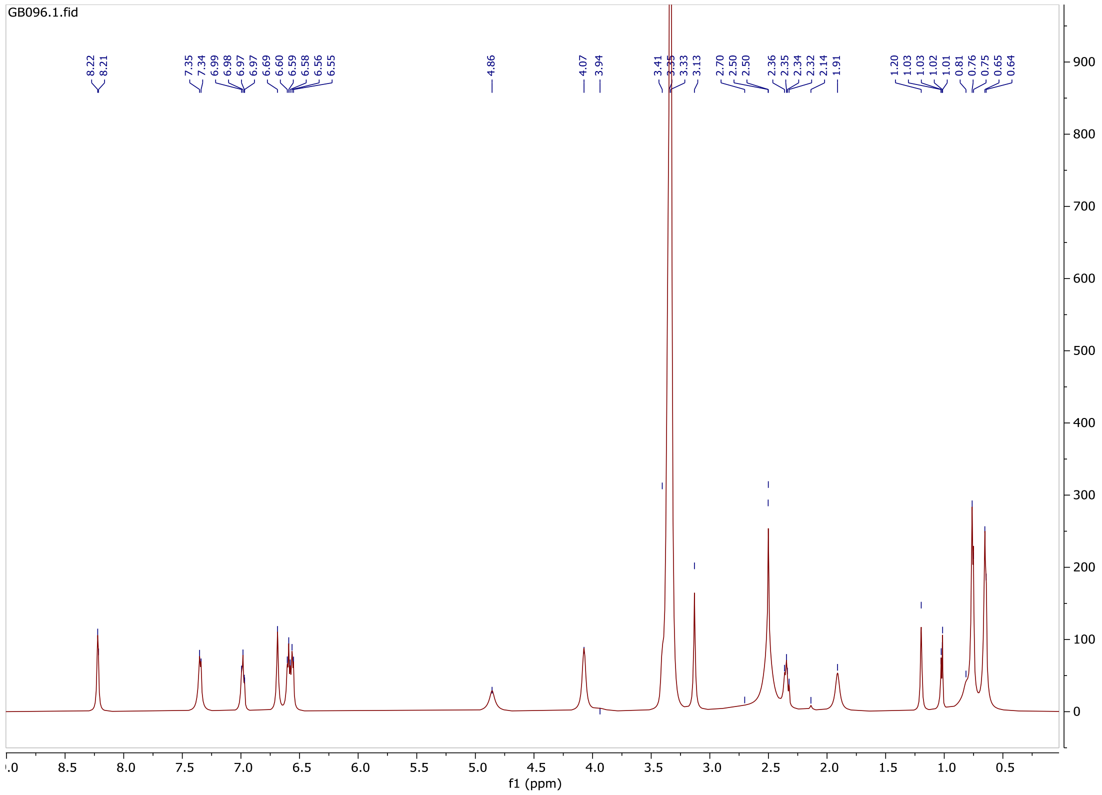

Figure S8 ^{1}H NMR spectrum of compound **3**.

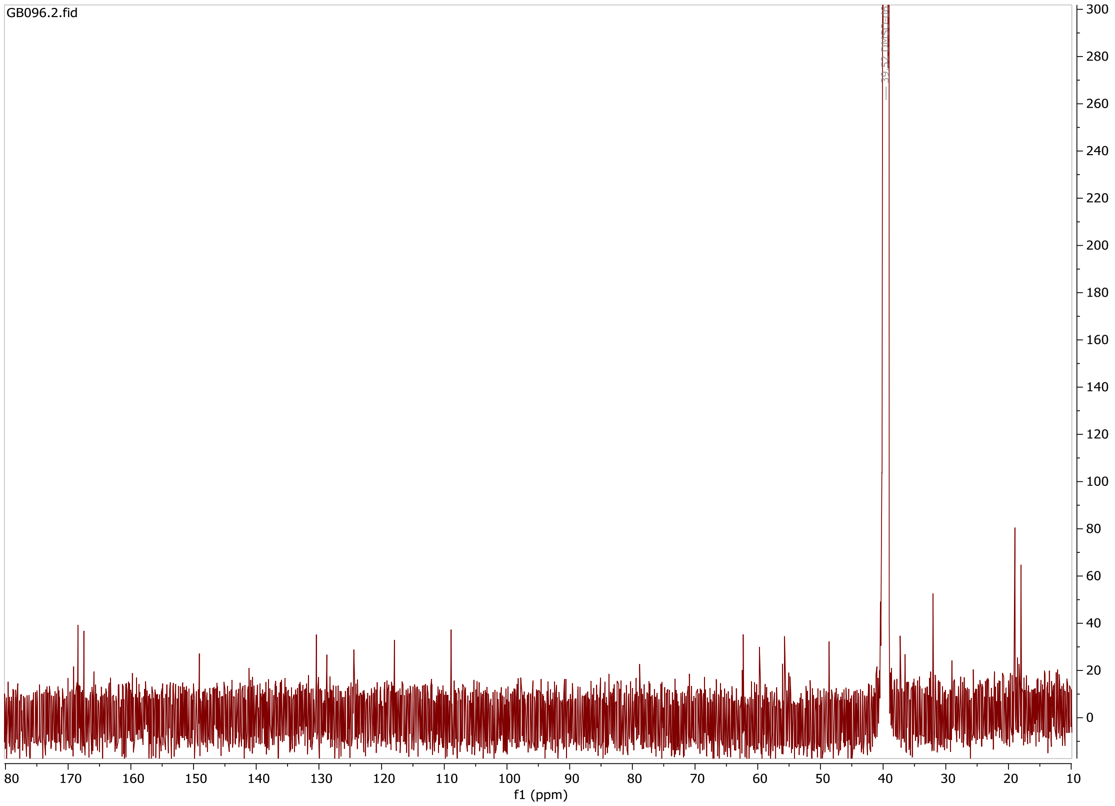

Figure S9 ^{13}C NMR spectrum of compound **3**.

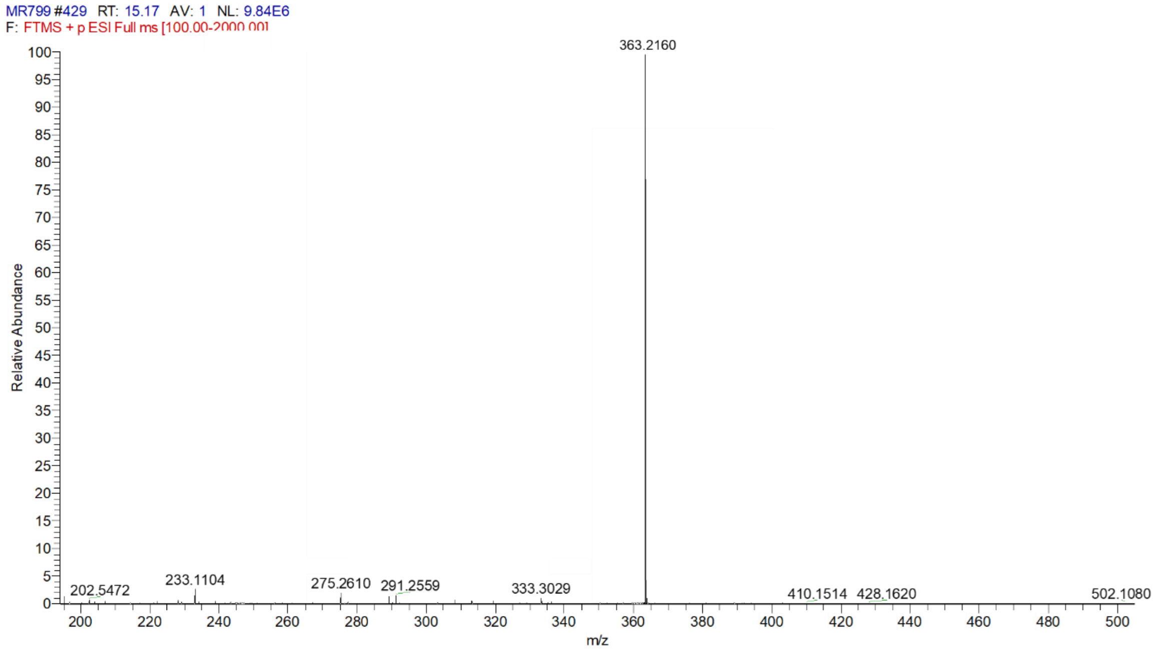

Figure S10 HRESIMS spectrum of compound **4**.

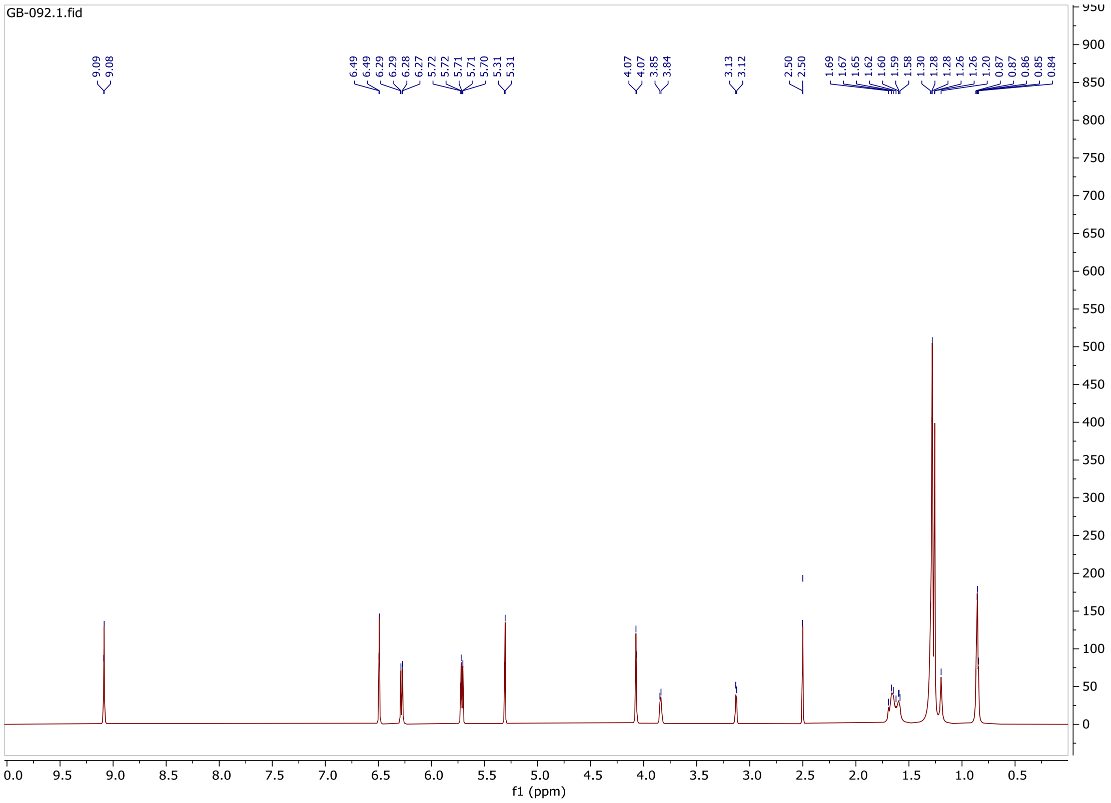

Figure S11 ^{1}H NMR spectrum of compound **4**.

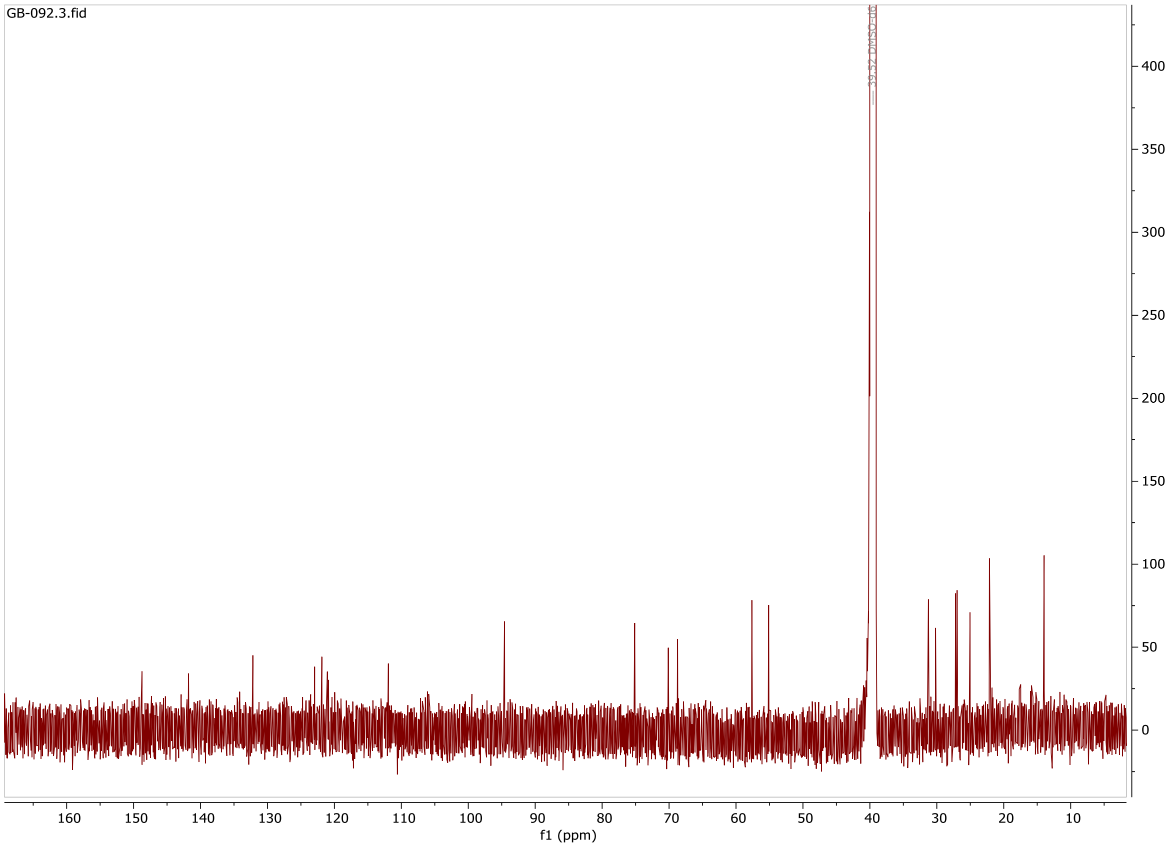

Figure S12 ^{13}C NMR spectrum of compound **4**.

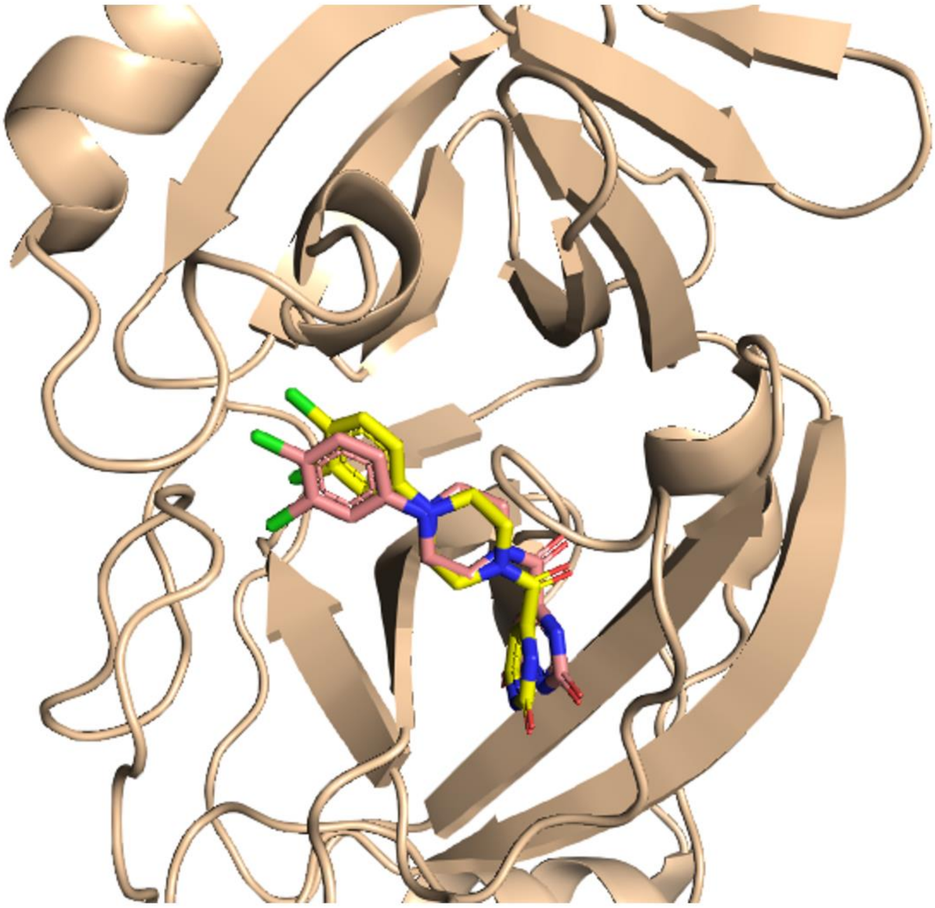

Figure S13. Binding modes of the co-crystalized ligand YD1 in its original and docking states inside the M^{pro} active site (RMSD = 1.1 Å).

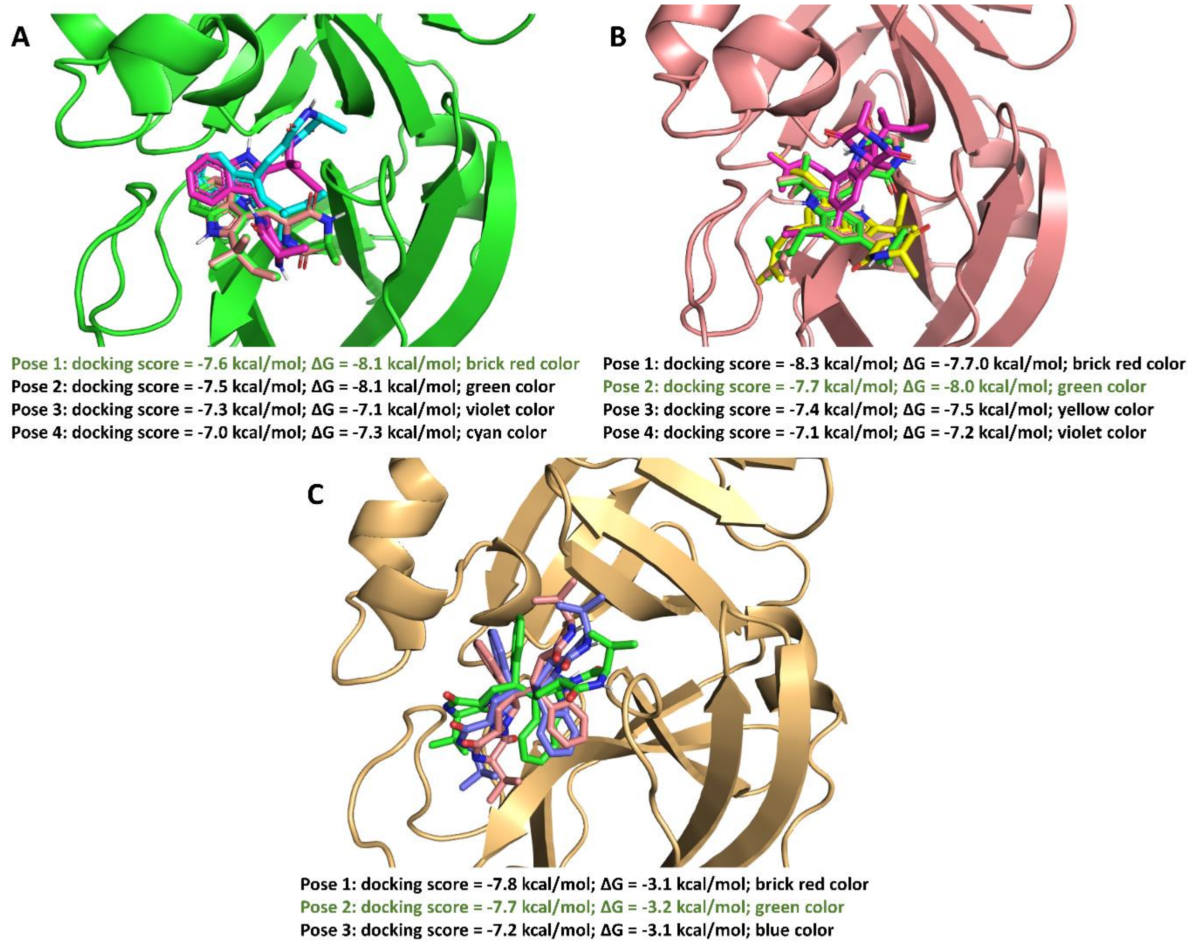

Figure S14. Different docking poses generated from the docking step of neoechinulin A (1), Echinulin (2), and eurocristatine (3) (A-C, respectively).

Methods

1. Docking and Molecular dynamic simulation

1.1. Ensemble Docking

AutoDock Vina software was used in all molecular docking experiments [1]. All isolated compounds were docked against the M^{pro} crystal structure (PDB codes: 7LTJ) [2]. The binding site was determined according to the enzyme's co-crystallized ligand. The co-ordinates of the grid box were: x = -12.87; y = 16.3; z = 68.64. The size of the grid box was set to be 10 Å. Exhaustiveness was set to be 24. Ten poses were generated for each docking experiment [3,4]. Docking poses were analysed and visualized using Pymol software [1].

1.2. Molecular Dynamics Simulation

Desmond v. 2.2 software was used for performing MDS experiments [5–7]. This software applies the OPLS-2005 force field. Protein systems were built using the System Builder option, where the protein structure was checked for any missing hydrogens, the protonation states of the amino acid residues were set (pH = 7.4), and the co-crystalized water molecules were removed. Thereafter, the whole structure was embedded in an orthorhombic box of TIP3P water together with 0.15 M Na^+ and Cl^- ions in 20 Å solvent buffer. Afterward, the prepared systems were energy minimized and equilibrated for 10 ns. For proteinligand complexes, the top-scoring poses were used as a starting points for simulation. Desmond software automatically parameterizes inputted ligands during the system building step according to the OPLS force field. For simulations performed by NAMD [8], the protein structures were built and optimized by using the QwikMD toolkit of the VMD software. The parameters and topologies of the compounds were calculated using the Charmm27 force field with the online software Ligand Reader and Modeler (http://www.charmm-gui. org/?doc=input/ligandrm, accessed on 16 April 2021) [9]. Afterward, the generated parameters and topology files were loaded to VMD to readily read the protein–ligand complexes without errors and then conduct the simulation step.

In regard to the SMD experiments, they were carried out by NAMD as described previously [8]. Comparison of the force profiles among different tested compounds was performed using constant-velocity SMD with a pulling rate of 0.025 Å/ps and with a spring constant of 7 kcal/mol/ $Å^2$. Several pulling velocities were preliminarily tested. We chose the one that gave the best balance between resolution among different ligands and simulation time length. The time length of the simulations was 1 ns, which was sufficient to observe the complete ligand unbinding. The mean force profile for each ligand was obtained by averaging the outcomes of three independent runs .the top-scoring poses were used as a starting points for simulation.

1.3. Absolute binding Free energy calculation

Binding free energy calculations (ΔG) were performed using the free energy perturbation (FEP) method [9]. This method was described in detail in the recent article by Kim and coworkers [9]. Briefly, this method calculates the binding free energy ΔGbinding according to the following equation: ΔGbinding = ΔGComplex - ΔGLigand. The value of each ΔG is estimated from a separate simulation

using NAMD software. All input files required for simulation by NAMD can be prepared by using the online website Charmm-GUI (https://charmm-gui.org/?doc=input/afes.abinding). Subsequently, we can use these files in NAMD to produce the required simulations using the FEP calculation function in NAMD. The equilibration (5 ns long) was achieved in the NPT ensemble at 300 K and 1 atm (1.01325 bar) with Langevin piston pressure (for "Complex" and "Ligand") in the presence of the TIP3P water model. Then, 10 ns FEP simulations were performed for each compound, and the last 5 ns of the free energy values was measured for the final free energy values [9]. Finally, the generated trajectories were visualized and analyzed using VMD software. It worth noting that Ngo and co-workers in their recent benchmarking study found that the FEP method of determination of ΔG was the most accurate method in predicting MPro inhibitors [10].

1.4. Drug-Likeness Analysis

Drug-like properties of the studied compounds were predicted by the commercially available software LigandScout 4.3 [11]. A list of SMILES codes of these compounds was prepared and submitted to the software to perform the drug-likeness calculations (e.g., molecular weight, hydrogen bond donors, hydrogen bond acceptors, number of rotatable bonds, topological polar surface area, and logP). As a final result, we checked if these calculated parameters for each compound followed Lipiniski' and Vebers' rules of drug likeness.

References

1. Seeliger, D.; de Groot, B.L. Ligand docking and binding site analysis with PyMOL and Autodock/Vina. J. Comput. Aided Mol. Des. 2010, 24, 417–422.

2. Clyde, A., Galanie, S., Kneller, D. W., Ma, H., Babuji, Y., Blaiszik, B., ... & Stevens, R. (2021). High Throughput Virtual Screening and Validation of a SARS-CoV-2 Main Protease Non-Covalent Inhibitor. *bioRxiv.*

3. Sayed, A.M.; Alhadrami, H.A.; El-Gendy, A.O.; Shamikh, Y.I.; Belbahri, L.; Hassan, H.M.; Abdelmohsen, U.R.; Rateb, M.E. Microbial natural products as potential inhibitors of SARS-CoV-2 main protease (Mpro). Microorganisms 2020, 8, 970

4. Amaro, R.E.; Baudry, J.; Chodera, J.; Demir, Ö.; McCammon, J.A.; Miao, Y.; Smith, J.C. Ensemble

docking in drug discovery. Biophys. J. 2018, 114, 2271–2278.

5. Bowers, K.J.; Chow, D.E.; Xu, H.; Dror, R.O.; Eastwood, M.P.; Gregersen, B.A.; Klepeis, J.L.; Kolossvary, I.; Moraes, M.A.; Sacerdoti, F.D.; et al. Scalable algorithms for molecular dynamics simulations on commodity clusters. In Proceedings of the SC’06: Proceedings of the 2006 ACM/IEEE Conference on Supercomputing, Tampa, FL, USA, 11–17 November 2006; IEEE: New York, NY, USA, 2006; p. 43.

6. Release, S. 3: Desmond Molecular Dynamics System, DE Shaw Research, New York, NY, 2017; Maestro-Desmond Interoperability Tools, Schrödinger: New York, NY, USA, 2017.

7. Schrodinger LLC. Maestro, Version 9.0; Schrodinger LLC: New York, NY, USA, 2009.

8. Phillips, J.C.; Braun, R.; Wang, W.; Gumbart, J.; Tajkhorshid, E.; Villa, E.; Chipot, C.; Skeel, R.D.; Kalé, L.; Schulten, K. Scalable molecular dynamics with NAMD. J. Comput. Chem. 2005, 26, 1781–1802.

9. Kim, S.; Oshima, H.; Zhang, H.; Kern, N.R.; Re, S.; Lee, J.; Rous, B.; Sugita, Y.; Jiang, W.; Im, W. CHARMM-GUI free energy calculator for absolute and relative ligand solvation and binding free energy simulations. J. Chem. Theory Comput. 2020, 16, 7207–7218.

10. Ngo, S.T.; Tam, N.M.; Quan, P.M.; Nguyen, T.H. Benchmark of Popular Free Energy Approaches Revealing the Inhibitors Binding to SARS-CoV-2 Mpro. J. Chem. Inf. Model. 2021, 61, 2302–2312.

11. Tutone, M.; Perricone, U.; Almerico, A.M. Conf-VLKA: A structure-based revisitation of the Virtual Lock-and-key Approach. J. Mol. Graph. Model. 2017, 71, 50–57.
